# Quality formation in *Peucedanum praeruptorum* dunn: metabolite biosynthesis, geoherbal variation, and early bolting regulation

**DOI:** 10.3389/fpls.2026.1812786

**Published:** 2026-04-28

**Authors:** Rubing Chen, Wei Wang, Cuiting Chen, Zhengyang Xu, Jinbao Pu, Pan Xu

**Affiliations:** 1 Tongde Hospital of Zhejiang Province, Hangzhou, China; 2 Zhejiang Academy of Traditional Chinese Medicine, Hangzhou, China; 3 Hangzhou Medical College, Hangzhou, China; 4 Zhejiang Chinese Medical University, Hangzhou, China

**Keywords:** coumarin biosynthesis, early bolting, geoherbal variation, *Peucedanum praeruptorum*, quality formation

## Abstract

*Peucedanum praeruptorum* Dunn is a traditional medicinal crop in which coumarins are the principal determinants of pharmacological efficacy and commercial quality. Evidence indicates that coumarin biosynthesis is governed by coordinated enzymatic and transcriptional regulation, with phenylpropanoid flux playing a central role in shaping the composition of major constituents. Regional variation in quality arises from the combined effects of environmental conditions, soil properties, and population differentiation, leading to distinct geoherbal characteristics. Early bolting represents a major constraint on cultivation, causing marked reductions in root coumarin content and quality. This process is closely associated with cultivation practices, particularly the use of first-year seeds and shortened growth cycles, together with environmental cues and developmental transitions, and is accompanied by root anatomical remodeling and reprogramming of phenylpropanoid metabolism, including enhanced lignification and altered metabolite allocation. Overall, medicinal quality in *P. praeruptorum* is determined by the interplay of metabolic regulation, environmental factors, cultivation practices, and developmental processes. These insights provide a mechanistic foundation for understanding quality formation and for developing more effective strategies to stabilize medicinal quality under cultivation.

## 1 Introduction

**Figure 1:**
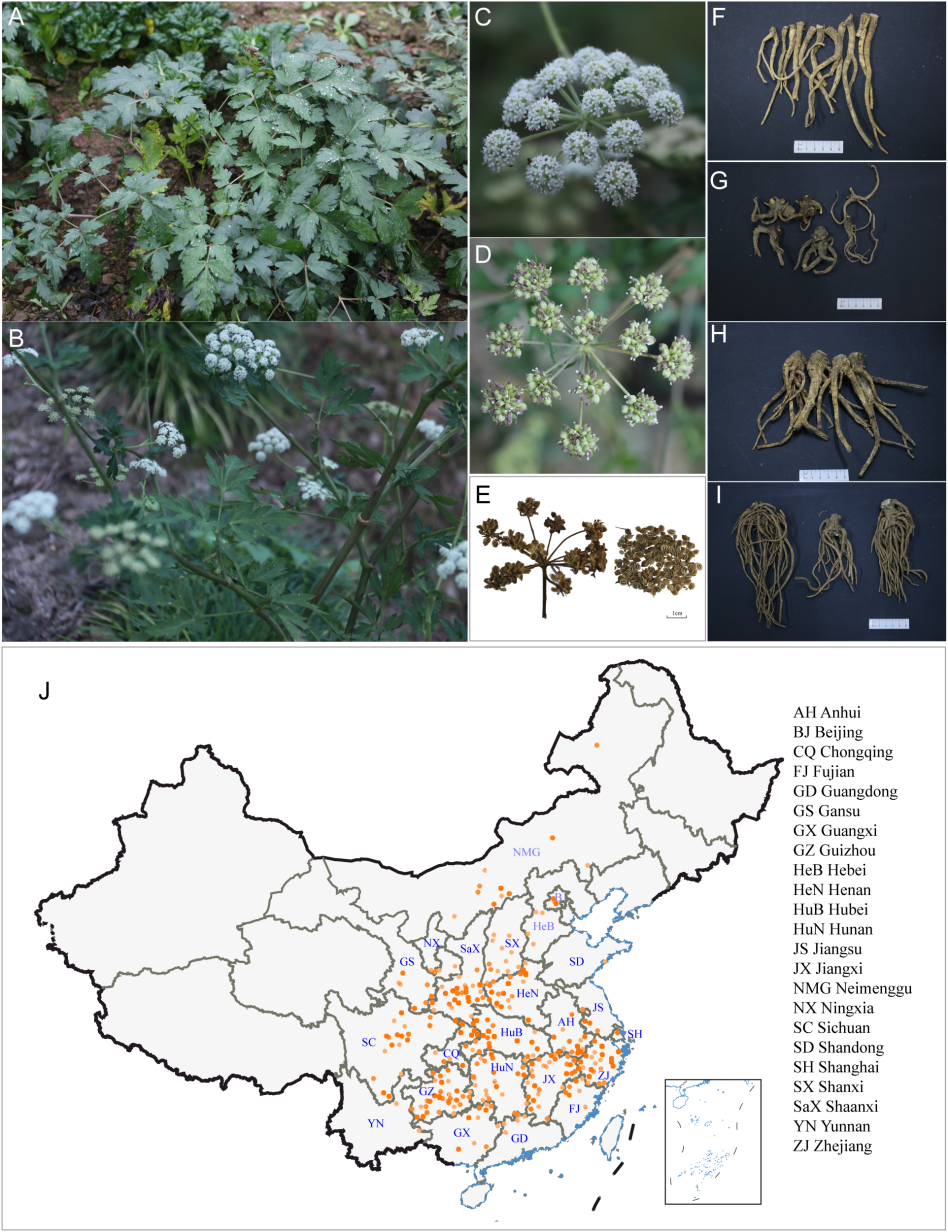
**(A)** Plant of unbolted *P. praeruptorum*; **(B, C)** Plant of bolted *P. praeruptorum*; **(D)** Fresh seeds; **(E)** Dried seeds **(F–I)** Dried roots; **(J)** Wild distribution of *P. praeruptorum* in China based on herbarium specimen records and published sources.

*Peucedanum praeruptorum* Dunn, a member of the Apiaceae family, is the botanical source of the traditional Chinese medicinal material known as *Qianhu*, which refers to its dried roots (Chu et al., 2020) (**Figure 1**). Its medicinal use dates back to the ancient text *Mingyi Bielu*, and the species typically inhabits forested mountain slopes at elevations of 250-2,000 m (Wang et al., 2024a). The core geoherbal region is centered in the Tianmu Mountain Range, spanning the border region of Zhejiang, Jiangxi, and Anhui provinces, particularly the areas of Chun’an (Zhejiang), Shangrao (Jiangxi), and Ningguo (Anhui). The local varieties, known respectively as *Chun Qianhu*, *Xin Qianhu*, and *Ning Qianhu* (Xu et al., 2023), are traditionally prized for thick, flexible roots, aromatic scent, and a distinctive flavor that begins sweet followed by bitterness and slight pungency (Ma et al., 2023). These traits have historically served as key indicators for quality assessment. In recent decades, large-scale cultivation has become the primary production mode, accompanied by the emergence of new producing regions across Fujian, Hubei, Hunan, Guizhou, Chongqing, Sichuan, and Yunnan (Wang et al., 2023).

Modern phytochemical studies have revealed that *P. praeruptorum* contains various secondary metabolites, including coumarins (Lee et al., 2015), volatile oils (Ma et al., 2023), and flavonoids (Tao et al., 2024). Among them, coumarins are recognized as the principal bioactive constituents, exhibiting anti-inflammatory (Yu et al., 2011), expectorant, antitussive, antiasthmatic (Lee et al., 2017), antithrombotic (Deng et al., 2022), and anticancer effects (Park et al., 2022; Wu et al., 2020). These pharmacological effects underscore the broad clinical potential of this medicinal plant.

As a biennial herb, cultivated *P. praeruptorum* typically undergoes vegetative growth in the first year, followed by reproductive development in the second. However, early bolting and flowering, which occurs during the first year of cultivation, disturb this developmental program, restricting the normal accumulation of secondary metabolites in the roots (Xie et al., 2023). Early bolting accelerates lignification and disrupts metabolite translocation such as coumarins, leading to inferior root texture and reduced active compound levels. Compounded by the depletion of wild resources, unstable quality in cultivated materials, and pronounced regional variation in medicinal traits, these challenges impede both yield improvements and quality standardization. Consequently, elucidating the molecular and metabolic mechanisms underlying these bottlenecks is essential for stabilizing production and ensuring consistent clinical efficacy.

Recent advances in genomics (Song et al., 2024b), transcriptomics (Bai et al., 2024; Song et al., 2022), and metabolomics (Song et al., 2017; Zhao et al., 2015) have greatly deepened the molecular understanding of *P. praeruptorum*. These studies have elucidated the coumarin biosynthetic pathway and its regulatory network (Huang et al., 2024; Liao et al., 2024), revealed the genetic and metabolic bases of quality divergence between wild and cultivated materials and among different producing regions, and begun to uncover the molecular mechanisms underlying geoherbalism (Li et al., 2025c) and early bolting (Song et al., 2022, Song et al., 2024a; Xie et al., 2023). Collectively, these findings outline a molecular framework that links genetic variation, environmental factors, and developmental processes to the formation/decline of medicinal quality. Nevertheless, systematic integration and practical application of these molecular insights remain limited.

Based on these advances, this review focuses on four interrelated themes central to quality formation in *P. praeruptorum*: (1) the chemical composition and distribution of constituents; (2) the biosynthesis and molecular regulation of coumarins and other related metabolites; (3) geographical origin identification and geoherbal quality, including quality variation between wild and cultivated materials and across various producing regions; and (4) the mechanisms and regulation of early bolting and flowering and their consequences on root quality. By integrating these findings, this review aims to clarify how these aspects collectively shape the medicinal quality of *P. praeruptorum*, thereby providing a scientific basis for improving quality control and advancing precision cultivation.

## 2 Chemical constituents and distribution patterns

### 2.1 Chemical constituents

#### 2.1.1 Coumarins

**Figure 2:**
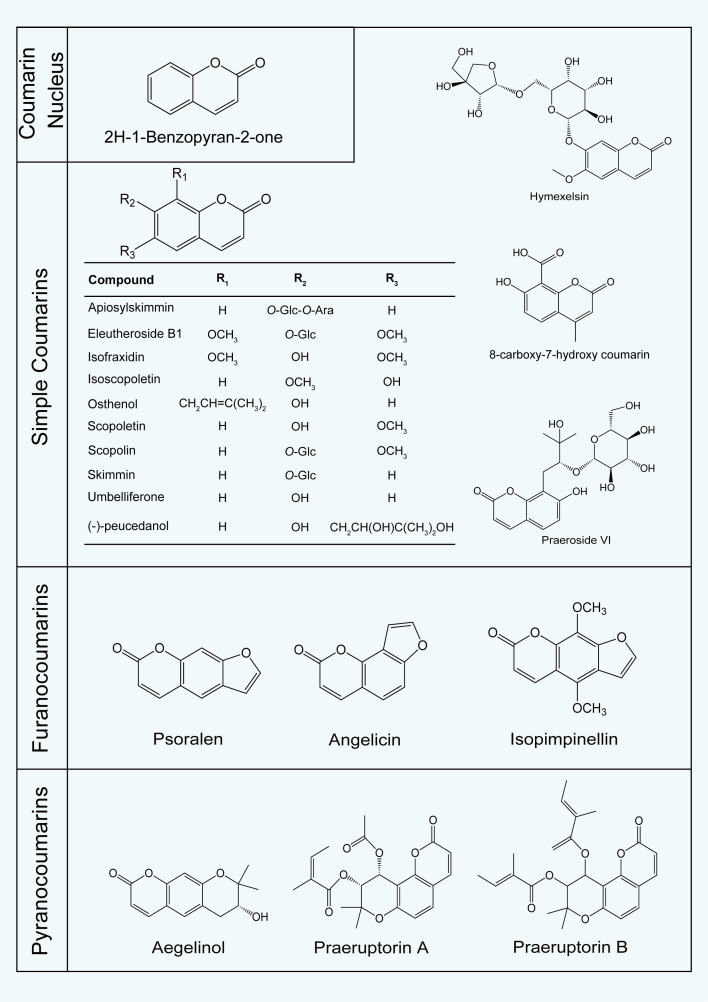
Representative structures of major coumarins identified in *P. praeruptorum*.

Coumarins represent the major class of chemical constituents in *P. praeruptorum* and share a characteristic benzo-α-pyrone backbone. Structurally, they can be broadly classified into furanocoumarins and pyranocoumarins, both of which include linear and angular subtypes. Advances in analytical techniques, particularly HPLC, MS, and NMR, have greatly facilitated the identification and structural characterization of diverse coumarins in *P. praeruptorum* (Chu et al., 2020; Hu et al., 2020; Lee et al., 2015). Representative structures are shown in **Figure 2**, and detailed information on individual coumarins is summarized in **Supplementary Table 1**.

Pyranocoumarins constitute the dominant subclass in *P. praeruptorum* (Huang et al., 2024). Among them, angular-type pyranocoumarins, including praeruptorin A (1.5–27.4 mg/g), praeruptorin B (0.2–15.0 mg/g), and praeruptorin E (0.106–7.687 mg/g), occur at relatively high levels and represent the principal bioactive constituents. In contrast, linear-type pyranocoumarins, such as pteryxin (up to 5.531 mg/g), are generally present at lower levels (Jiang et al., 2026). Notably, praeruptorin A and praeruptorin B are specified as key quality control markers for *P. praeruptorum* in the Chinese Pharmacopoeia.

Furanocoumarins are typically present at lower levels. Linear-type furanocoumarins, including imperatorin (0.000–1.188 mg/g), bergapten (0.000–0.471 mg/g), and psoralen (0.000–0.058 mg/g), are more frequently reported, whereas angular-type furanocoumarins, such as oxypeucedanin (0.002–0.340 mg/g), occur at similarly low levels. Most of these compounds are present at concentrations below 1–2 mg/g. Simple coumarins are minor constituents and typically occur only in trace amounts. For example, umbelliferone (0.006–0.136 mg/g) and isofraxidin (0.000–0.150 mg/g) are present at much lower levels compared with pyranocoumarins (Chen et al., 2019; Jiang et al., 2026).

#### 2.1.2 Volatile oils

**Figure 3:**
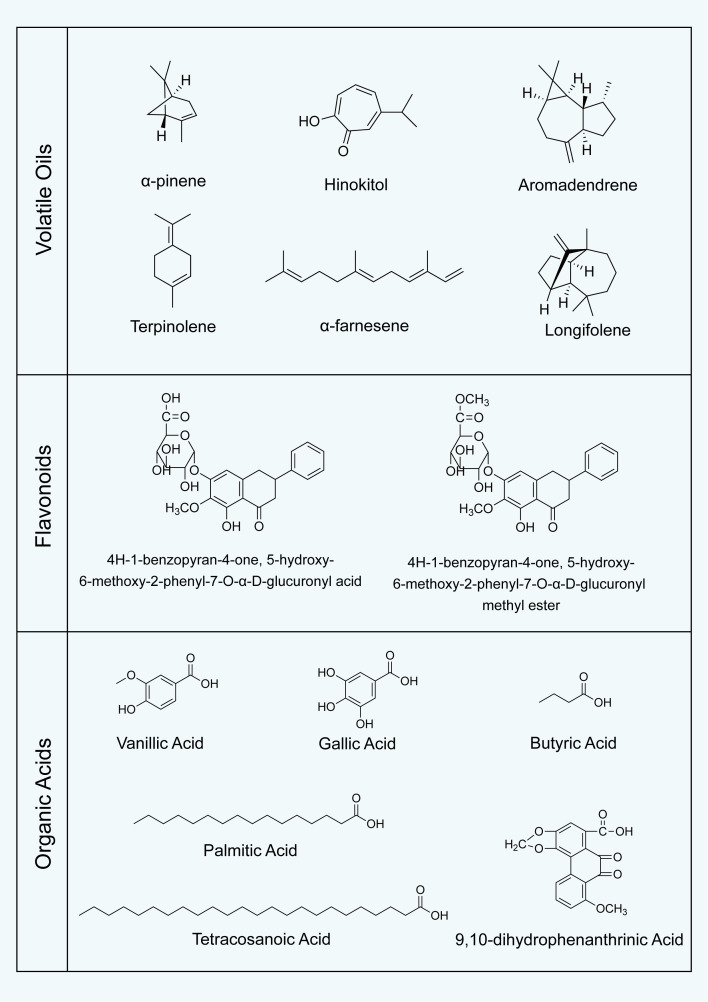
Major volatile oil constituents, flavonoids, and organic acids identified in *P. praeruptorum*.

Volatile oils represent another important class of constituents in *P. praeruptorum*, although their overall abundance is lower than that of coumarins. These constituents are primarily terpenoids, including monoterpenes, sesquiterpenes, and their oxidized derivatives. The volatile profile exhibits pronounced regional variation. For instance, samples collected from Zhejiang, Anhui, and Jiangxi contain 21 identified volatile constituents, among which α-pinene, hinokitol, aromadendrene, terpinolene, α-farnesene, and longifolene are the dominant components (**Figure 3**), together accounting for more than 60% of the total volatile fraction (Yu et al., 2007). In contrast, samples from Fujian contain 38 volatile constituents, representing 77.97% of the total volatile content, with β-phellandrene, α-bisabolol, and β-pinene as the major components (Chen et al., 2002).

More recently, headspace gas chromatography-ion mobility spectrometry (HS-GC-IMS) has enabled the characterization of a fingerprint comprising approximately 80 volatile constituents. When combined with principal component analysis (PCA), this approach allows efficient discrimination of samples harvested at different periods, with those collected in December exhibiting a particularly rich and diverse volatile profile (Ma et al., 2023). However, the pharmacological activities and biosynthetic origins of most volatile constituents remain poorly understood.

#### 2.1.3 Flavonoids and other constituents

Flavonoids have also been identified in *P. praeruptorum*. The first reported flavonoids comprised 4H-1-benzopyran-4-one, 5-hydroxy-6-methoxy-2-phenyl-7-O-α-D-glucuronyl acid, and its methyl ester derivative (Zhang et al., 2012). In addition to flavonoids, several organic acids have been characterized (Chen et al., 2021), including vanillic acid, gallic acid (Kong et al., 1994), butyric acid (Zhang et al., 2009), palmitic acid, tetracosanoic acid (Zhang et al., 2006), and 9,10-dihydrophenanthrinic acid (Zhang et al., 2010). Recent studies also report the presence of polysaccharides and other classes of secondary metabolites (Zhang et al., 2023a; Zhao et al., 2022). These constituents remain relatively less investigated, and their biological roles and pharmacodynamic relevance not yet well understood. Further studies are required to elucidate their potential contributions to the overall medicinal quality.

### 2.2 Distribution patterns

The biosynthesis and accumulation of coumarin metabolites in *P. praeruptorum* exhibit pronounced temporal and spatial specificity, and these patterns are closely linked to the formation of medicinal quality. The composition and content vary dynamically during plant development (Jia et al., 2024). During the vegetative growth stage, the concentrations of major active constituents in the roots increase steadily and remain relatively high and stable in winter, which is regarded as the optimal harvest season (Nie et al., 2013; Xing, 2023; Yu et al., 2013). Once the reproductive development begins, metabolic activity shifts from storage and accumulation to the formation of reproductive structures, resulting in a general decline in root coumarin levels. Multi-regional cultivation trials further suggest that harvesting between November and the following February ensures high and stable total coumarin content while maintaining desirable yields, thereby maximizing economic benefit (Tan et al., 2024).

In addition to temporal variation, the distribution of secondary metabolites exhibits pronounced spatial specificity. Metabolic profiles in stems and leaves often resemble those of the roots, and in certain samples, praeruptorin A levels in aerial parts even exceeded those in roots, indicating their potential as alternative sources of these compounds (Kong, 2010). As development progresses, differentiated allocation patterns emerge: praeruptorin E is preferentially enriched in leaves, praeruptorin B accumulates chiefly in roots, while praeruptorin A remains relatively abundant in both tissues. During the reproductive phase, however, all three compounds tend to accumulate in the stems, accompanied by a pronounced decrease in root content (Li, 2022).

At the tissue level, coumarins are predominantly enriched in regions outside the vascular cambium, such as the periderm, cortex, and phloem, with much lower levels in the secondary xylem (Xie et al., 2020). Spatial metabolomics has further refined this distribution pattern, revealing strong mass spectrometry imaging (MSI) signals in the periderm, cortex, and phloem of roots (Li and Li, 2024). Laser-capture microdissection combined with MSI further demonstrated that structurally related coumarins maintain similar spatial distribution patterns before and after bolting, suggesting regulation by homologous gene networks (Chen et al., 2019). Notably, these spatial and temporal variations in coumarin distribution are closely associated with developmental changes in root anatomy, further supporting an intrinsic link between tissue structure and metabolite accumulation. Current studies remain at the organ and tissue levels, and the precise subcellular localization, storage forms of coumarin, and spatial distribution of minor components have yet to be elucidated.

## 3 Biosynthesis and regulation of coumarins

Coumarin biosynthesis represents the core metabolic process underlying the formation of medicinal quality in *P. praeruptorum*. Omics analyses have greatly advanced the understanding of this pathway, revealing how enzymatic modules, modification processes and regulatory networks interact to generate both the quantity and structural diversity of coumarins. A clear mechanistic framework is essential for interpreting chemical differences among producing regions and identifying molecular targets relevant to quality formation. This section outlines the major biosynthetic steps, structural modification processes, regulatory mechanisms and transport features associated with coumarin formation.

### 3.1 Formation of umbelliferone via the phenylpropanoid pathway

**Figure 4:**
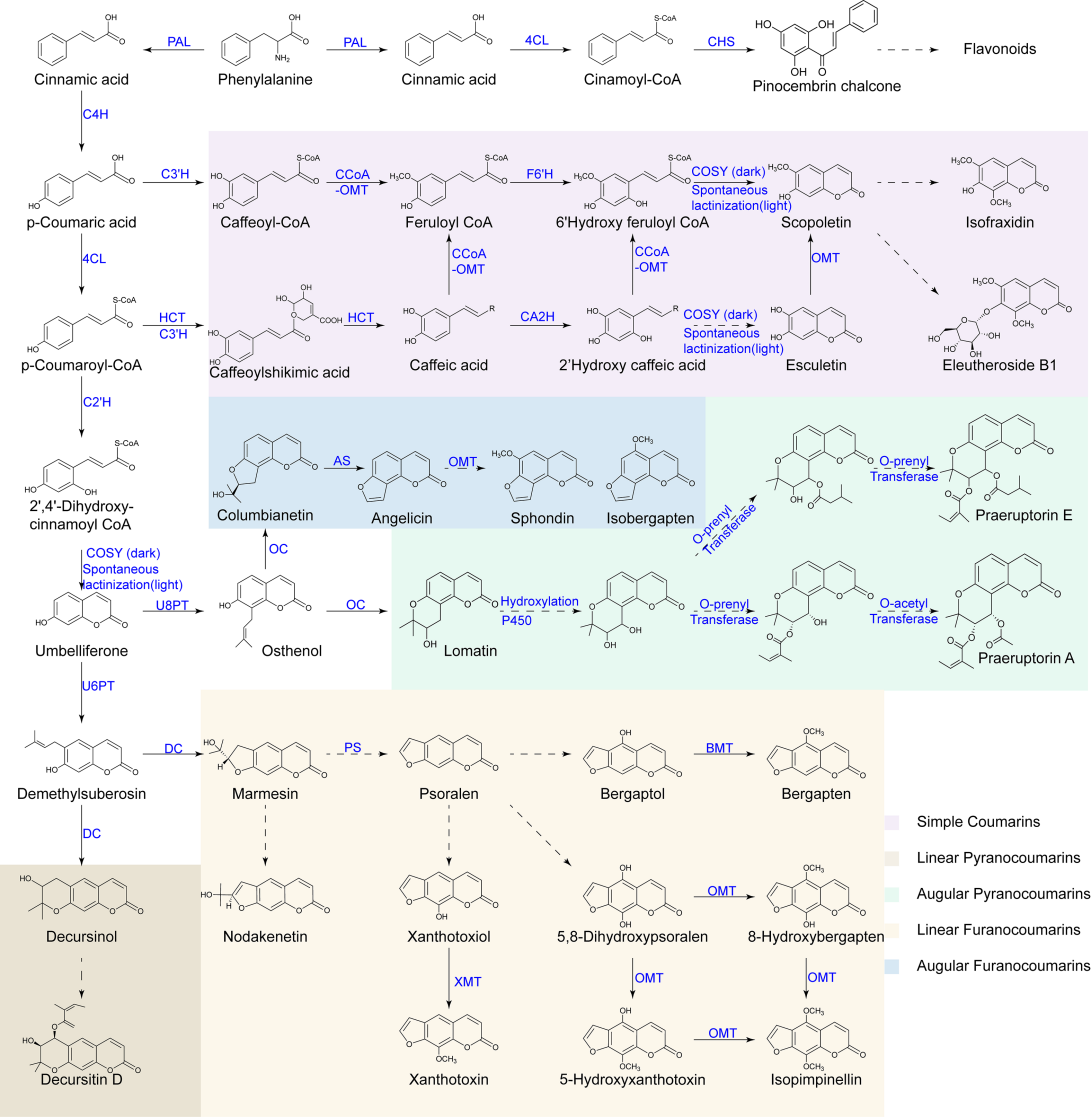
Coumarin biosynthetic pathway in *P. praeruptorum*. Solid arrows indicate enzymatic reactions that have been experimentally characterized, whereas dashed arrows represent putative or multi-step conversions that remain to be fully elucidated. Enzymes are shown in blue.

Coumarin biosynthesis originates from the conserved phenylpropanoid pathway and involves precursor formation, branch-determining hydroxylation, and lactone-ring formation (**Figure 4**). Phenylalanine is converted to cinnamic acid by phenylalanine ammonia-lyase (PAL) (Sui et al., 2019). Cinnamic acid is then hydroxylated by cinnamate 4-hydroxylase (C4H) to produce *p*-coumaric acid (Wang et al., 2020b), which is subsequently converted to *p*-coumaroyl-CoA by 4-coumarate CoA ligase (4CL) (Liu et al., 2017).

At the branching hydroxylation step, *p*-coumaroyl-CoA 2’-hydroxylase (C2’H) catalyzes the ortho-hydroxylation of *p*-coumaroyl-CoA to form an ortho-hydroxylated intermediate (Yao et al., 2017). This intermediate undergoes cis-trans isomerization and lactonization mediated by O-hydroxycinnamoyl-CoA-dependent coumarin synthase (COSY), resulting in the formation of umbelliferone, the core precursor for most complex coumarins (Kim et al., 2023; Vanholme et al., 2019). A parallel branch mediated by feruloyl-CoA 6’-hydroxylase (F6’H) acts on feruloyl-CoA and, together with COSY, produces scopoletin, a representative simple coumarin (Yao et al., 2017).

Upstream biosynthetic genes involved in precursor formation exhibit functional differentiation. Among the three *4CL* isoforms, *4CL1* shows the highest affinity for p-coumaric acid and ferulic acid, indicating its predominant role in this module (Liu et al., 2017). Analyses support *C4H* identity as a CYP73A enzyme co-expressed with other structural genes (Zhao et al., 2015), catalyzing the conversion of cinnamic acid to p-hydroxycinnamic acid, confirming its typical *C4H* activity (Wang et al., 2020b).

*C2’H* plays a decisive role in directing metabolic flux toward complex coumarins and has been cloned and its function verified by *in vitro* enzymatic assays (Yao et al., 2017). The telomere-to-telomere genome assembly of *P. praeruptorum* has further revealed gene colocation and family expansion around *C2’H*, supporting coordinated regulation within this module (Bai et al., 2024). Consistent evidence from *Saposhnikovia divaricate* shows that overexpression *SdC2’H* significantly promotes coumarin accumulation (Su et al., 2025).

Although *F6’H* has not been biochemically confirmed, multiple studies repeatedly annotated it as a core enzyme in the simple coumarin branch (Zhao et al., 2015). *F6’H* and genes involved in lactone ring formation are arranged as gene clusters on chromosomes and are co-expressed with several members of the *myeloblastosis* transcription factor family (Liao et al., 2024). Moreover, *F6’H* is hypothesized to have evolved from *C2’H* (Huang et al., 2024). In the related Apiaceae species such as *Heracleum moellendorffii*, *Cnidium monnieri* and *Angelica dahurica*, *F6’H* acts as a rate-limiting step responsive to pathogen infection (Liu et al., 2024; Shi et al., 2020; Zhang et al., 2023d), which leads to increased accumulation of simple coumarins.

Lactone-ring formation is catalyzed by COSY, which is essential for avoiding intermediate accumulation and maintaining coumarin productivity. In *Arabidopsis thaliana*, *COSY* knockout leads to a marked reduction of coumarin levels (Vanholme et al., 2019). Subsequent structural and mechanistic studies revealed that *COSY* is a BAHD-type acyltransferase family (BAHD) facilitating cis-trans isomerization and ring closure (Kim et al., 2023). Its genomic colocation with other biosynthetic genes in *P. praeruptorum* supports a conserved pathway organization (Bai et al., 2024).

### 3.2 Biosynthetic modification of complex coumarins

Following the umbelliferone formation, complex coumarins are synthesized through a sequence of prenylation, side-chain cyclization, and terminal modification steps. Membrane-bound prenyltransferases (PT) of the UbiA superfamily catalyze the prenylation of umbelliferone to generate either the linear intermediate demethylsuberosin (DMS) or the angular intermediate osthenol (Zhao et al., 2024). Three *PTs* including *PT1*(*U6PT*), *PT2*(*U8PT*), and *PT3* have been identified in *P. praeruptorum*, with amino acid residues Thr161 and Ala161 determining regiospecific substitution at the C-6 or C-8 position (Huang et al., 2024; Zhao et al., 2024). These *PTs*, together with *C2’H* and osthenol cyclase (*OC*), are located within a gene cluster on the same chromosome and thus form a biosynthetic gene cluster responsible for complex coumarin biosynthesis.

After prenylation, side-chain cyclization is catalyzed by decursinol cyclase (DC) and OC, whose functional specificity is determined by Glu303 and Asp301, respectively (Huang et al., 2024). DC uses DMS to yield linear intermediates such as marmesin and decursinol, whereas OC converts osthenol into angular intermediates including columbianetin and lomatin (Jian et al., 2020b). *CYP71AJ* enzymes then catalyze oxidative reactions to form psoralen in the linear branch. In the angular pathway, angelicin synthase (AS), also belonging to the *CYP71AJ* family, catalyzes the formation of angelicin. Ultraviolet (UV) treatment enhances *CYP71AJ* expression, thereby increasing the metabolic flux toward psoralen and angelicin synthesis.

Terminal modification mediated by O-methyltransferase (OMTs) and BAHD acyltransferases generate major coumarins such as praeruptorin A, B, and E (Bai et al., 2024; Zhao et al., 2016). In *P. praeruptorum*, several candidate OMTs have been identified. Among them, bergaptol O-methyltransferase (BMT) catalyzes the methylation of bergaptol to form bergapten. Kinetic and substrate recognition analyses have further confirmed that hydroxylation precedes methylation in this pathway. Further studies elucidated the site selectivity and substrate specificity of caffeic acid O-methyltransferase (COMT) toward bergaptol and xanthotoxol (Zhao et al., 2019). The expression pattern of *BMT* parallels the accumulation of furanocoumarin derivatives and is markedly upregulated by methyl jasmonate (MeJA). These features indicate that *BMT* plays a key role in methylation-level modification.

In parallel with *OMT*-mediated modification, UDP-glycosyltransferase (*UGT*) genes form co-expression networks with structural genes of the coumarin biosynthetic pathway under bolting and various stress conditions (Song et al., 2024b, Song et al., 2023), and their expression is enriched in roots and secretory canals. Although the specific enzymatic functions of *UGT* and *BAHD* unique to *P. praeruptorum* have not yet been experimentally validated, their expression levels show coordinated changes with coumarin accumulation under inductive treatments. Accordingly, *UGT* and *BAHD* are considered components of the proposed complete coumarin biosynthetic pathway. This arrangement indicates that the post-modification module may represent an important regulatory node.

### 3.3 Multilevel regulatory networks underlying coumarin biosynthesis

Coumarin biosynthesis in *P. praeruptorum* is modulated by a regulatory framework in which transcriptional control, external hormonal treatments, biotic stress response and plant-microbe interactions collectively shape pathway activity.

Transcription factors play crucial roles in regulating coumarin biosynthesis. Among the regulators identified so far, R2R3-MYB transcription factors such as *MYB3* and *MYB103* show expression profiles that correlate closely with the accumulation of praeruptorin A and B. Their activity is consistent with roles as positive regulators that activate key structural genes (Liao et al., 2024). Genome-scale analyses further reveal that several key biosynthetic genes, *PT*, *OC*, and *C2’H*, are collocated within a gene cluster, and show coordinated expression across tissues, implying that physical gene clustering can facilitate coordinated transcriptional responses within this pathway.

Coumarin metabolism also displays pronounced responsiveness to hormonal and environmental cues (Wan et al., 2024). Exogenous phytohormones such as MeJA can enhance the transcription of *C2’H* and *PAL* in roots. At appropriate concentrations, melatonin can upregulate key pathway genes, thereby promoting growth and development while simultaneously increasing the synthesis of coumarin compounds. Moreover, the first rate-limiting enzyme in the pathway, PAL, responds strongly to MeJA, UV light, temperature stress, and hydrogen peroxide (H_2_O_2_) (Sui et al., 2019), and its transcript level correlates with coumarin accumulation, highlighting the environmental sensitivity of upstream flux control. Consequently, exogenous signals including hormones, light, and temperature modulate transcription factors and the promoters of metabolic genes, thereby enabling *P. praeruptorum* to dynamically adjust coumarin biosynthesis in response to environmental fluctuations.

Microorganisms also constitute important biological components of the regulatory network governing coumarin biosynthesis (Liu et al., 2022; Song et al., 2025). Root-associated microorganisms such as *Penicillium restrictum*, isolated from the roots of *P. praeruptorum*, can trigger plant defense-related signaling cascades that produce novel coumarins during fermentation, indicating that microbial activity can enrich the structural diversity of coumarins (Wang et al., 2024b). Field inoculation experiments further show that *P. restrictum* reduced early bolting rate while concurrently elevating coumarin accumulation in roots, consistent with microbe-induced defense responses that reprogram the coumarin biosynthetic pathway (Song et al., 2025).

### 3.4 Transport and redistribution of coumarins

Intracellular transport and inter-organ redistribution contribute to the spatial patterning of coumarins in *P. praeruptorum*. Although specific transporters have not yet been biochemically identified in this species, transcriptomic evidence suggests that multidrug resistance (MDR) and ATP-binding cassette (ABC) transporter families participate in the sequestration and movement of coumarins and their glycosylated forms (Song et al., 2022). Similar mechanisms have been described in *Arabidopsis*, where the ABC transporter ABCG37 mediates root efflux of coumarin derivatives (Fourcroy et al., 2014). Cytochrome P450 enzymes also facilitate tissue-to-tissue movement of partially modified coumarins (Zhao et al., 2015). These transport processes provide an additional layer of regulation shaping organ-specific accumulation.

## 4 Quality variation between wild and cultivated *P. praeruptorum*

### 4.1 Morphological and phytochemical differentiation

With the gradual depletion of wild resources, artificial cultivation has become the primary method of providing *P. praeruptorum*. The transition from wild harvesting to large-scale cultivation has revealed pronounced differences in both root morphology and phytochemical accumulation. Wild roots typically are irregular in shape with sparse branching, dense annular rings near the crown, lighter epidermal color, sparse lenticels, fragile texture, and indistinct oil spots on the fracture surface (Wang et al., 2024c). In contrast, cultivated roots are thicker and more robust, with dense branching, wider ring spacing, more numerous and larger lenticels, and distinctly visible oil spots (Chu et al., 2020; Zou et al., 2025). These macroscopic features not only aid in the morphological identification of the medicinal material but also reflect differences in growth cycles, developmental history, and growth environment between wild and cultivated populations.

Microscopic observations further highlight this divergence. The transverse sections of wild roots show a thicker cork layer, abundant oil cavities in the phloem, dense xylem vessels, and strong birefringence under polarized light, whereas cultivated roots contain fewer oil cavities and vessels (Zou et al., 2025), features that correlate negatively with coumarin content. Consistent with these anatomical traits, the contents of praeruptorin A, B, and E are generally higher in wild populations than in cultivated ones (Chu et al., 2020; Liang et al., 2014; Shi et al., 2022). Cultivated materials often exhibit greater fluctuations and overall lower levels than wild ones. Nonetheless, some studies report only minor differences (Wang and Chen, 2009), and others even exhibited higher constituent levels in cultivated samples (Nie et al., 2013). These discrepancies are largely attributable to differences in cultivation regions, growth duration, and harvest periods. Accordingly, quality differences cannot be explained by cultivation status alone, and the underlying mechanisms require further clarification.

### 4.2 Mechanisms of quality divergence in cultivated *P. praeruptorum*

**Figure 5:**
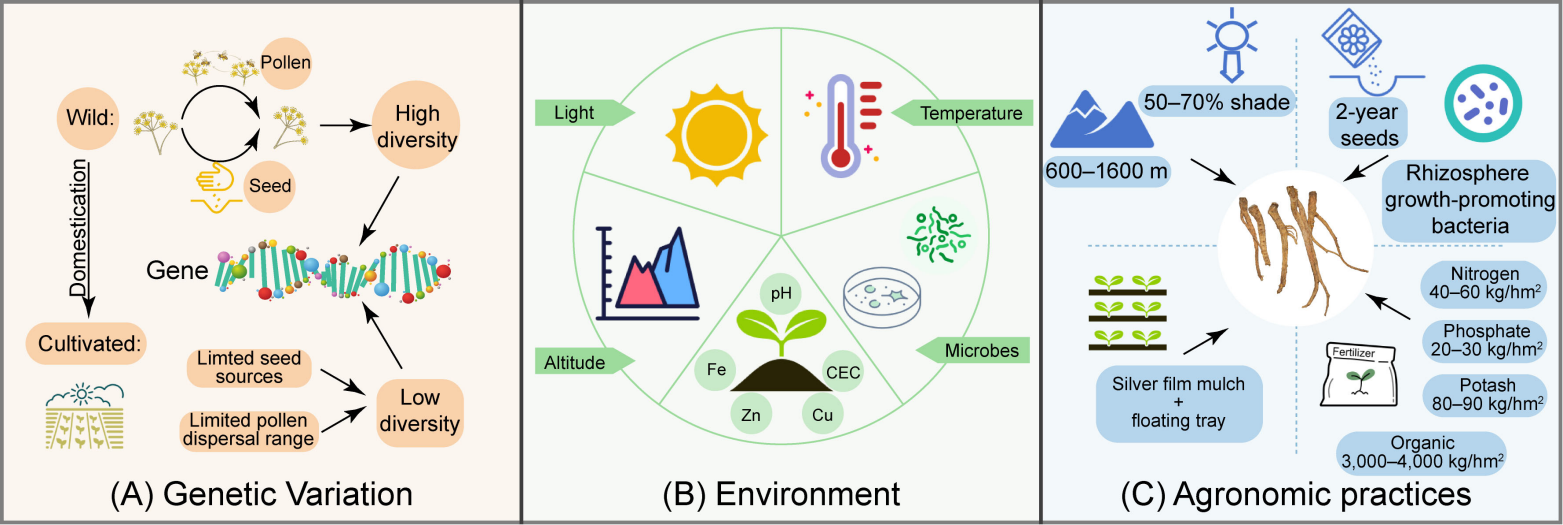
Schematic representation of key factors influencing the quality of cultivated *P. praeruptorum*. **(A)** Genetic variation; **(B)** Environmental factors; **(C)** Agronomic practices that improve root development and coumarin accumulation.

Quality decline in cultivated *P. praeruptorum* results from the combined effects of genetic, environmental, and agronomic practices. Among these, constraints arising from current production practices, particularly early bolting, are increasingly recognized as key contributors, as they directly shorten the vegetative growth period and limit root development, reserve accumulation, and coumarin enrichment. Meanwhile, genetic background and environmental conditions continue to modulate the stability and variability of these processes (**Figure 5**).

Premature transition to reproductive growth compresses the time available for root enlargement and metabolite accumulation, thereby weakening the biosynthesis and storage of coumarins and other quality-related constituents. This constraint may be further reinforced by the repeated use and successive propagation of first-year seeds, which can gradually reduce germplasm quality and destabilize growth performance over time. Excessive fertilization, particularly under high nitrogen and phosphorus input, may promote rapid vegetative growth and increase bolting risk, ultimately compromising the quality stability of cultivated materials (Qu et al., 2022; Rao et al., 2025).

Environmental conditions further influence the quality of cultivated P. praeruptorum (**Figure 5B**). Factors such as temperature (Xu et al., 2021c), light intensity, altitude (Xu et al., 2023), endophytic microorganisms (Liu et al., 2022), and soil physicochemical properties (Liang et al., 2021) collectively regulate plant growth and secondary metabolism. At the same time, cultivation practices associated with current production systems may indirectly contribute to changes in genetic structure. The widespread use of first-year seeds and long-term successive propagation without systematic germplasm renewal may reduce genetic variability at the population level, while the shortening of the growth cycle caused by early bolting may further affect the stability of cultivated germplasm. This trend is supported by molecular analyses, which including clustering and PCA, have revealed clear differentiation between wild and cultivated populations, with lower polymorphism levels observed in cultivated materials (Pan et al., 2024; Zhou et al., 2021). These changes may collectively lead to genetic homogenization, which could reduce adaptive flexibility under heterogeneous cultivation environments and increase susceptibility to environmental and management-related stresses. In this context, genetic factors are more appropriately viewed as outcomes of long-term cultivation practices that may further exacerbate the instability of medicinal quality, rather than as independent primary drivers of short-term quality variation.

### 4.3 Strategies for improving the quality of cultivated materials

In response to these challenges, several environmental and cultivation strategies have been developed to improve the quality of cultivated *P. praeruptorum* (**Figure 5C**). Optimizing cultivation conditions, such as cultivation at altitudes of 900-1,200 m combined with approximately 60% shading, has been shown to suppress early bolting and enhances coumarin accumulation (Xu et al., 2023). Application of rhizosphere growth-promoting bacteria can modulate the endophytic microbial community and thereby promote the accumulation of secondary metabolites (Luo et al., 2024; Zhang et al., 2025).

In addition, improvements in cultivation practices are essential. Agronomic practices such as silver film mulching and floating-tray seedling transplantation improve yield and cultivation efficiency (Zheng et al., 2019). Meanwhile, rational fertilization strategies are also critical; optimized fertilization regimes, particularly with appropriate phosphorus and potassium, have been shown to promote coumarin biosynthesis, whereas excessive nitrogen input should be avoided (Chen et al., 2020; Dou, 2018; Guo et al., 2024).

Despite these advances, current evidence remains fragmented, and a comprehensive mechanistic framework linking genetic variation, ecological conditions, developmental regulation and agronomic practices to coumarin biosynthesis is still lacking. A coordinated approach that brings together multi-platform analytical technologies, environmental modeling and field-based cultivation experiments will be essential for developing targeted strategies to restore and stabilize the quality of cultivated *P. praeruptorum*.

## 5 Molecular basis of geoherbal quality in *P. praeruptorum*

### 5.1 Regional differentiation and origin identification

**Figure 6:**
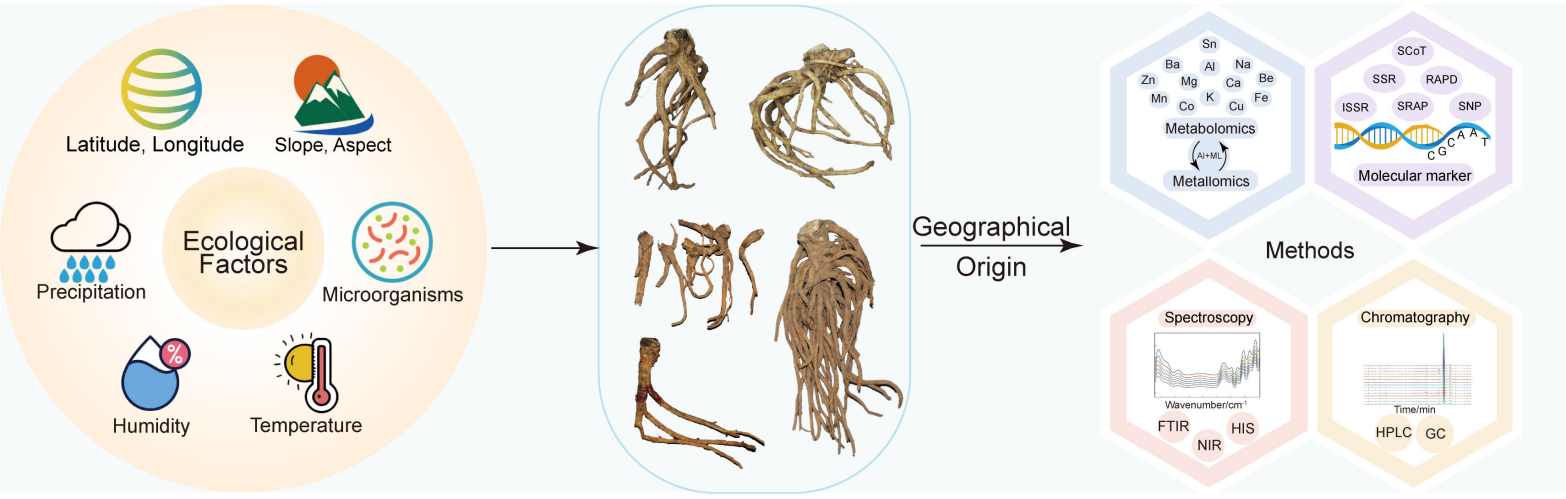
Conceptual framework for the formation of geoherbal quality (left) and identification of geographical origin (right) in *P. praeruptorum*.

Geoherbal medicines are traditionally distinguished by characteristic morphological traits and superior intrinsic quality. However, origin authentication based solely on morphology remains empirical and is therefore prone to substantial subjectivity. The accuracy of such identification is easily affected by cultivation age, harvest time, and postharvest handling, and consistent morphological criteria for origin discrimination are still lacking (Li et al., 2020). Consequently, recent research has increasingly shifted toward analytical approaches that are based on quantifiable, reproducible and objective data (**Figure 6**).

Near-infrared spectroscopy (NIRS), combined with chemical fingerprinting and chemometric modeling, provides a rapid and non-destructive approach for discriminating samples from different production regions (Hu et al., 2019; Lou et al., 2020). However, the application range of this model remains limited, and the generalization of NIRS-based models requires further validation. Meanwhile, chemical fingerprinting coupled with pattern recognition methods, including PCA and orthogonal partial least squares-discriminant analysis (OPLS-DA), has led to the establishment of an analytical framework centered on coumarin constituents. This analytical approach is sufficiently robust for origin discrimination yet flexible enough to support population-level quality evaluation (Li et al., 2025b; Ni et al., 2025b; Xie et al., 2024; Xu et al., 2021b).

With growing datasets and improved analytical workflows, geoherbalism studies have begun incorporating inorganic element fingerprints alongside key organic metabolites to construct a dual-dimensional quality evaluation system. This strategy substantially improves both the accuracy and cross-regional applicability in origin authentication (Li et al., 2025a). On this basis, a predictive model that couples inorganic element fingerprints with organic metabolite profiles has been developed (Li et al., 2025a), enabling geographical traceability of *P. praeruptorum* through joint analysis of selected elements (Mn, Ca, Mg) and specific coumarin derivatives.

Molecular markers strengthen a complementary line of evidence for origin authentication through spatial genetic structure. Simple sequence repeat (SSR) markers mined from RNA-seq datasets have greatly enhanced resolution, identifying more than 12,000 SSR loci and generating over 8,000 primer pairs, of which approximately 800 exhibit high polymorphism and stability (Shi et al., 2017). Chloroplast genome sequencing further enabled the identification of cpSSR loci and highly variable haplotypes with clear regional patterns (Li et al., 2019). Moreover, inter-simple sequence repeat (ISSR) and sequence-related amplified polymorphism (SRAP) have consistently revealed distinct genetic clustering among populations from Zhejiang, Jiangxi, and Anhui, showing a positive correlation with geographical distance (Zhou et al., 2021).

### 5.2 Quality control of geoherbal *P. praeruptorum*

Geographical differentiation in the quality of *P. praeruptorum* arises from the translation of environmental heterogeneity into metabolic divergence (**Figure 6**). Climate factors, light intensity, terrain features, soil properties, and rhizosphere microbiota modulate key steps in the phenylpropanoid pathway, producing distinct regional profiles of coumarins and other secondary metabolites (Ni et al., 2025a). Ecological regionalization studies using geographic information systems (GIS) and the maximum entropy (MaxEnt) modeling identified precipitation as the primary determinant of habitat suitability. Optimal production areas are mainly concentrated in the border regions of Hubei, Hunan, and Chongqing, as well as the mountainous regions where Zhejiang, Jiangxi, and Anhui converge (Wu et al., 2021). Cluster analysis and grey correlation analysis have further revealed that average annual humidity and summer temperature exert the strongest influence on coumarin content, whereas light exposure, precipitation, and winter temperature contribute to a lesser degree (Xu et al., 2021c). Collectively, these findings suggest that humid climates with moderate summer temperatures provide favorable conditions for quality formation.

Topographic variables such as latitude, longitude, slope and aspect exhibit limited direct explanatory power (Lou et al., 2023), and their effects likely operate indirectly through modulating light distribution and precipitation gradients. Recent studies on microbial communities have isolated several endophytic fungi from *P. praeruptorum*, among which fungi *Chaetomium* spp. can synthesize coumarins and, when introduced into the rhizosphere, suppress early bolting without compromising coumarin accumulation (Liu, 2022; Ma et al., 2021; Zhang et al., 2023b, Zhang et al., 2023c). However, current work remains preliminary, particularly regarding strain identification, functional verification, and stability of microbial interventions under field conditions. Rigorous mechanistic studies are still needed to clarify how environmental cues are perceived, unified, and transduced into metabolic outputs that define geoherbal quality.

## 6 Molecular mechanisms and control of early bolting and flowering

Under practical cultivation conditions, some *P. praeruptorum* plants are susceptible to early bolting before the optimal harvest season, even when they have been sown for less than one year. It causes profound changes in root development, metabolic allocation, and secondary metabolite accumulation, ultimately leading to the decline of medicinal quality. This section consolidates current understanding of the phenotypic impacts, environmental and cultivation drivers, and molecular regulatory mechanisms underlying early bolting and flowering.

### 6.1 Effects of early bolting on the medicinal quality

#### 6.1.1 Anatomical alterations of roots

Anatomical evidence indicates that early bolting significantly alters the morphology and tissue organization of *P. praeruptorum* roots (Song et al., 2016). In unbolted plants, root tissues progress from the periderm through the pericycle parenchyma to the secondary phloem, vascular cambium, and secondary xylem. In early-bolted plants, however, the proportional areas of the pericycle parenchyma and secondary phloem are markedly reduced, whereas the secondary xylem expands substantially and exhibits pronounced lignification of vessels and fibers (Xie et al., 2023, Xie et al., 2020). These changes may be associated with a reallocation of metabolic flux within the phenylpropanoid pathway, with flux preferentially directed toward lignin biosynthesis at the expense of coumarin production. This metabolic shift likely contributes both to increased lignification and to the observed decline in coumarin content, thereby linking anatomical remodeling with changes in secondary metabolism and medicinal quality.

#### 6.1.2 Changes in chemical constituents

Multiple studies have demonstrated that early bolting markedly reduces the levels of root coumarins, although the magnitude of decline varies among reports (Chen and Han, 2013). For instance, the contents of praeruptorin A, praeruptorin B, and praeruptorin E decreased by 26.6%, 52.1%, and 30.3%, respectively (Song et al., 2022), with praeruptorin B showing the most pronounced decline. However, some studies have reported increases in specific compounds such as praeruptorin I and II (Song et al., 2022), and others reported divergent trends or no significant change between praeruptorin A and B after early bolting (Li et al., 2022c; Xie et al., 2020). Despite these discrepancies, most evidence indicates that early bolting disrupts the coumarin profile in roots, typically manifested as a substantial reduction in praeruptorin B, ultimately compromising medicinal quality. These variations among studies likely stem from differences in geographical origin, cultivation practices, and the developmental timing of bolting, highlighting the central role of environmental factors in determining the chemical quality of the herb.

#### 6.1.3 Physiological and biochemical reprogramming

The transition from vegetative to reproductive growth induces extensive metabolic reprogramming in Apiaceae family (Li et al., 2022b). During the reproductive stage, cell division in meristematic tissues intensifies, organelle numbers increase, and levels of free amino acids and soluble sugars rise, reflecting heightened overall metabolism. *P. praeruptorum* leaves exhibit significant changes in soluble protein, polysaccharides, and antioxidant enzyme activities, such as peroxidase (POD) and superoxide dismutase (SOD) during bolting (Yang, 2024). These findings indicate that bolting consumes large amounts of nutrients, shifts the metabolic focus toward reproductive development, and weakens the biosynthesis of active constituents in roots, ultimately contributing to quality decline.

### 6.2 Factors influencing early bolting and corresponding control strategies

#### 6.2.1 Cultivation factors and strategies

##### 6.2.1.1 Seed selection and sowing schedule optimization

Seed maturity and quality are major determinants of the early bolting rate. Seeds collected from fully mature umbels, sun-dried to 9–11% moisture, exhibit significantly reduced early bolting incidence (Zhang et al., 2021). The timing of seed harvesting is equally critical. When the seed pericarp has turned yellow-green and collected at the first year after sowing, it can show a lower bolting rate (Xu et al., 2023).

Sowing time involves a trade-off between yield and early bolting risk. In Chongqing, sowing before mid-January resulted in bolting rate above 40%, while sowing in March reduced it to below 10% (Xu et al., 2021a). However, sowing after late February also caused a marked decline in yield. Similarly, trends were observed in Guizhou (Jian et al., 2020a). Regional climate should therefore guide sowing schedules, with late February to early March generally recommended.

##### 6.2.1.2 Fertilization measures and agronomic practices

Fertilization plays a key role in modulating early bolting. Soil conditions, especially excessive nitrogen and phosphorus inputs, may predispose plants to early bolting by promoting rapid vegetative growth, altering root-shoot nutrient allocation, and enhancing root lignification. Field studies recommend applying organic fertilizer together with 45% potassium sulfate compound fertilizer before sowing, followed by fertilization management tailored to plant developmental stages (Wang et al., 2020a). The commonly adopted principle “adequate fertilization in early growth, moderate control in mid-growth, and stimulation at later stages” effectively balances vegetative and reproductive growth. Over-fertilization or premature application of compound fertilizers can trigger the reproductive transition; therefore, fertilization should be applied based on soil fertility and rainfall patterns. Empirical evidence suggests that fertilizing before light rain or after heavy rain enhances nutrient uptake and reduces the likelihood of early bolting (Du et al., 2022).

Other agronomic practices can also help delay bolting. Semi-wild cultivation practices (Du et al., 2021; Wang et al., 2023), such as using seeds collected from perennial wild populations, intercropping on marginal land, and implementing timely pruning or topping, have been shown to effectively suppress early bolting. Shading is another commonly employed agronomic practice. Production trials in *P. praeruptorum* have demonstrated that moderate shading (approximately 60-70%) effectively reduces early bolting rate while maintaining both coumarin content and overall yield (Jiang et al., 2026; Xu et al., 2023). These results indicate that appropriate agronomic practices, including semi-wild cultivation, pruning, intercropping, and shading, provides practical and effective means for suppressing early bolting in *P. praeruptorum*.

#### 6.2.2 Environmental factors and strategies

Temperature, light, and altitude are the primary environmental drivers of early bolting in *P. praeruptorum*. Among these factors, light plays a particularly important role in coordinating plant development and secondary metabolism. Field observations indicate that cultivation under strong light conditions, such as on dry, sun-exposed slopes, is associated with an increased risk of early bolting (Qiu et al., 2010), whereas moderate shading (60-70%) can effectively delay the transition to reproductive growth. Light intensity not only affects bolting behavior but also influences the coumarin accumulation. Recent studies have shown that increasing shading levels lead to a progressive decline in the contents of bergapten, praeruptorin A, and praeruptorin E, while praeruptorin B exhibits an opposite trend. These differential responses indicate that individual coumarin components are distinctly regulated by light conditions (Xie et al., 2026).

Altitude also influences early bolting and quality formation. Cultivation at elevations of 900–1200 m is associated with lower bolting rates and higher coumarin content, indicating that moderate ecological conditions are favorable for both growth and metabolite accumulation (Xu et al., 2023). Soil physicochemical properties may also influence early bolting in *P. praeruptorum*. In addition, rhizosphere microbiome communities may indirectly affect plant development by modulating nutrient availability and root growth. Collectively, these findings indicate that environmental factors regulate early bolting and medicinal quality through coordinated effects on plant growth and metabolite accumulation.

### 6.3 Molecular mechanisms of early bolting and flowering

**Figure 7:**
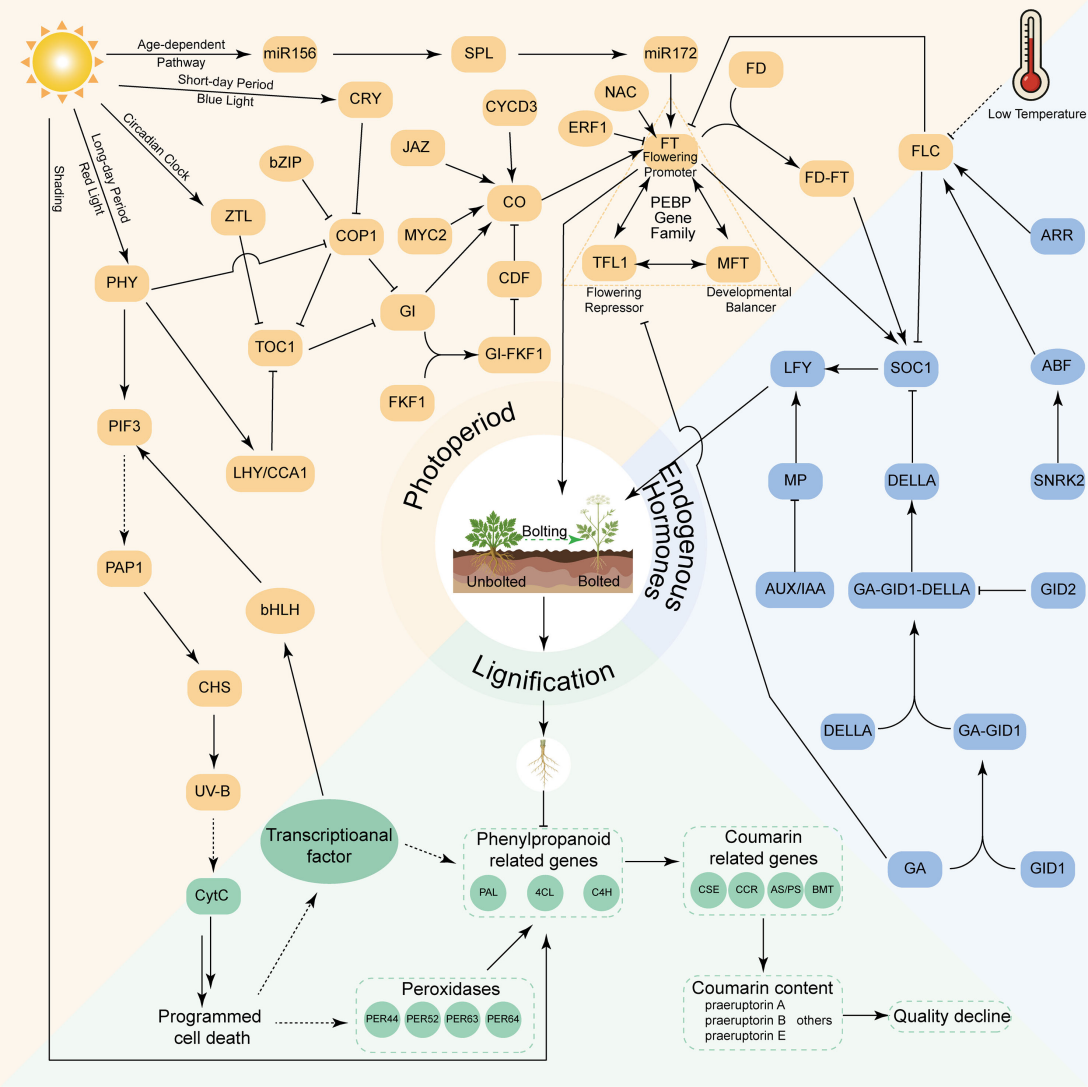
Molecular mechanisms of early bolting and flowering in *P. praeruptorum*. Arrows indicate promotion, whereas blunt-ended lines indicate inhibition. Solid lines indicate regulatory or signaling relationships supported by experimental evidence, whereas dashed lines denote indirect, inferred, or as-yet unvalidated interactions or processes.

Early bolting in *P. praeruptorum* arises from the coordinated action of environmental cues, endogenous hormonal pathways, and extensive transcriptional reprogramming. With the application of omics technologies, a clearer mechanistic framework has begun to emerge, revealing how photoperiodic signaling, floral transition pathways, and secondary metabolic networks intersect to drive early bolting and the associated decline in medicinal quality (**Figure 7**).

#### 6.3.1 Photoperiod

Photoperiodic regulation provides the primary environmental input controlling bolting. Light signals perceived by the photoreceptors phytochrome (PHY) and cryptochrome (CRY) are integrated into the circadian system, where components such as zeitlupe (ZTL) and gigantea (GI) relay photic information to the central flowering pathway. This regulatory cascade converges on the constans (CO)-flowering locus T (FT) module, which function as a key molecular switch for the transition from vegetative growth to reproductive development. The activity of CO is further coordinated with the age-dependent microRNA156 (miR156)-squamosa promoter-binding protein-like (SPL) pathway, ensuring that floral induction occurs only when developmental competence is achieved (Song et al., 2021; Xie et al., 2023). Recent studies under graded shading conditions have provided additional support for the central role of photoperiodic regulation by identifying candidate genes associated with early bolting that respond to changes in light intensity (Xie et al., 2026).

Transcriptomic analyses have identified multiple regulators associated with circadian rhythm and photoperiodic responses in *P. praeruptorum*, providing species-specific molecular support for this regulatory framework (Song et al., 2021). In addition to floral induction, photoperiodic signaling appears to coordinate developmental and metabolic processes. PHY-mediated light perception, partly through interaction with phytochrome-interacting factor 3 (PIF3), may link circadian regulation with phenylpropanoid and coumarin biosynthetic pathways, thereby coupling light sensing to secondary metabolic reprogramming during bolting.

In contrast, the role of vernalization in *P. praeruptorum* remains unclear due to the lack of direct experimental evidence. While vernalization-dependent flowering has been well characterized in related Apiaceae species such as *A. sinensis*, where the flowering locus C (FLC)–mediated pathway plays a central role (Fan et al., 2022), comparable evidence has not yet been established in *P. praeruptorum*. Current evidence suggests that early bolting in this species is primarily regulated by photoperiodic and light-responsive pathways, which may operate largely independently of vernalization.

#### 6.3.2 Endogenous hormonal regulation

Endogenous hormones precisely regulate the bolting and flowering process of *P. praeruptorum* through coordinated interaction networks. Gibberellins (GAs) act as core signaling molecules, initiating the Ga-Gibberellin Insensitive Dwarf 1 (GID1)-DELLA signaling cascade that triggers degradation of DELLA proteins via the ubiquitin-proteasome pathway (Gao and Fu, 2018; Huang et al., 2015). Perception of bioactive GAs by the GID1 receptor leads to ubiquitin-proteasome-mediated degradation of DELLA proteins, thereby releasing repression on downstream floral regulators and enabling floral meristem differentiation in the shoot apical meristem (Xie et al., 2023).

Furthermore, analysis of conserved domains in key enzymes of *P. praeruptorum* revealed that the 2OG-FeII_Oxy dioxygenase domain in *C2’H* exhibits catalytic activity related to GA biosynthesis (Li, 2022). Although direct biochemical evidence is still lacking, this homology raises the possibility of crosstalk between GA metabolism and coumarin biosynthetic processes. Collectively, these findings suggest that endogenous hormones function as central mediators linking developmental signaling and environmental stress responses, playing a pivotal role in the reproductive transition of *P. praeruptorum*.

#### 6.3.3 Metabolic-developmental crosstalk

Following the environmental and hormonal signaling described above, early bolting in *P. praeruptorum* is accompanied by coordinated transcriptional reprogramming across tissues (Song et al., 2021, Song et al., 2023). This reprogramming integrates floral-transition pathways with lignification processes and coumarin metabolism, indicating a close coupling between developmental progression and secondary metabolic regulation. Several core flowering regulators from the phosphatidylethanolamine-binding protein (PEBP) gene family, including *FT*, *FT1*, and *MFT*, are likely involved in bolting-associated developmental transitions (Song et al., 2024a). Consistent with this coordinated regulation, transcriptional changes in photoperiod- and circadian-related components coincide with the induction of *cauliflower 1* (*CAL1*) and *cauliflower 2* (*CAL2*) in both leaves and roots, supporting activation of floral meristem identity pathways and their coordination with subsequent metabolic and anatomical changes (Guan et al., 2023).

Concomitant with reproductive transition, genes associated with lignification and cellular remodeling, including *peroxidase* (*PRX*) and *autophagy-related genes* (*ATG*), are upregulated after bolting (Song et al., 2023), implying enhanced lignin biosynthesis and secondary cell-wall thickening. Within the R2R3-MYB transcription factor family, *MYB3* and *MYB54* are associated with activation key phenylpropanoid pathways genes, whereas *MYB15* and *MYB61* are more specifically associated with bolting-related lignification (Liao et al., 2024). Notably, the brassinosteroid signaling kinase gene, *brassinosteroid insensitive 2* (*BIN2*) shows high expression in xylem tissue, suggesting that hormone-linked signaling components involved in vascular differentiation and secondary growth participate in the xylem expansion and lignification characteristic of early-bolted roots (Yang et al., 2024).

These structural changes are accompanied by a marked, organ-dependent redistribution of coumarin biosynthesis during reproductive development (Li, 2022). Expression of key biosynthetic genes, including *4CL*, *C2’H*, and *COMT-S*, decline sharply in roots and stems, while homologous genes are broadly induced in leaves after bolting. Meanwhile, the sharp reduction of *BMT* expression in stems during flowering implies that late-stage tailoring reactions may become limiting as plants transition into the reproductive phase (Wei et al., 2023). The concurrent upregulation of ABC transporter family genes is consistent with strengthened interorgan transport and likely contributes to reduced root constituent accumulation. Emerging evidence further suggests that this metabolic reprogramming involves light-responsive regulatory processes. Several genes in the phenylpropanoid pathway, including *4CL*, *cinnamoyl-CoA reductase* (*CCR)*, *caffeoyl shikimate esterase* (*CSE*), and *caffeoyl-CoA O-methyltransferase* (*CCoAOMT*), are associated with coumarin accumulation, implying that regulatory networks may influence the partitioning of metabolic flux between lignin formation and coumarin biosynthesis (Xie et al., 2026).

Together, these co-expression patterns and inferred regulatory relationships support a close association between early bolting, reprogrammed phenylpropanoid metabolism, intensified cell-wall formation, and declining medicinal quality. However, most current evidence remains correlative, underscoring the need for targeted functional assays to establish causal links among these processes.

#### 6.3.4 Epigenetic mechanisms

Epigenetic regulation has emerged as an additional layer shaping bolting and downstream physiological programs. Evidence from model plant *A. thaliana* indicates that DNA methylation can influence flowering time by modulating the expression of flowering regulators (Kim et al., 2014; Li et al., 2022a; Sun et al., 2022). In *A. sinensis*, genome-wide DNA methylation increases after bolting and flowering (Yuan et al., 2024), and demethylation can promote flowering under long-day conditions in *Perilla frutescens* (Kondo et al., 2010). Beyond developmental timing, DNA methylation also participates in regulating specialized metabolite accumulation by altering methylation states at key biosynthesis loci. For example, promoter CHH methylation after bolting has been associated with enhanced formation of ferulic acid and lignin precursors in *A. sinensis* (Yuan et al., 2024). Hypomethylation of pathway genes affects platycodin biosynthesis in *Platycodon grandiflorus* (Kim et al., 2020). To date, epigenetic regulation has not been reported in *P. praeruptorum*. The roles of DNA methylation in coumarin biosynthesis and in quality variation related to early bolting remain unclear.

Significant progress has been made in elucidating the molecular networks through which environmental factors and endogenous hormones regulate bolting and flowering. However, the causal relationship between early bolting and coumarin biosynthesis, as well as the specific molecular mechanisms by which environmental cues exert their effects, remains insufficiently understood.

## 7 Conclusions and perspectives

In conclusion, research on *P. praeruptorum* has shifted from cataloging chemical constituents toward understanding how medicinal quality is formed and destabilized under cultivation. Recent advances have clarified key regulatory steps in metabolite biosynthesis (Huang et al., 2024), geoherbal variation (Li et al., 2025c), and early bolting (Song et al., 2021, Song et al., 2024a; Xie et al., 2023), providing a more integrated view of quality formation and its vulnerability during expanded cultivation. These studies support a unified framework in that genotype, environment, cultivation practice, and developmental stage jointly shape coumarin profiles and medicinal quality. Future research can advance along three convergent directions: resolving coumarin metabolism across tissues and developmental stages, explaining field variation under realistic environments, and controlling early bolting to ensure yield and quality stability.

A central priority is to develop a comprehensive understanding of coumarin metabolism, encompassing biosynthesis, subcellular organization, storage, and transport in relation to medicinal quality. The spatial organization and intracellular handling of coumarins remain largely unresolved, representing a major barrier to understanding how metabolic processes are coordinated with tissue structure and function. Addressing this challenge will require integrative approaches, including spatial metabolomics, cell-type- or single-cell-resolved transcriptomics, and targeted functional validation. Such efforts will be essential to clarify how these processes are coordinated across cellular and developmental contexts, and how this coordination underpins the formation and stability of medicinal traits. From an applied perspective, a deeper mechanistic understanding of these regulatory and transport processes will provide a foundation for more precise and controllable manipulation of coumarin composition.

For large-scale production, achieving stable medicinal quality requires a clearer understanding of the sources of variation observed under realistic cultivation conditions. Rather than focusing solely on genetic determinants, future research should emphasize how environmental factors, cultivation regimes, and developmental processes interact with metabolic regulation to shape coumarin profiles and quality traits. Clarifying these interactions will be essential for explaining the variability of geoherbal characteristics and for developing more reliable strategies to maintain quality consistency under field conditions.

Early bolting remains a major limitation to yield and quality stability under cultivation. Key priorities include identifying dominant environmental triggers under typical cultivation regimes and elucidating the regulatory mechanisms through which hormone signaling, gene expression and epigenetic regulation coordinate the floral transition. Understanding how bolting is coupled with shifts in phenylpropanoid metabolism and root lignification will provide a basis for integrated control strategies involving germplasm selection, cultivation management, and rhizosphere regulation. Overall, these directions provide a mechanistic foundation for standardized quality evaluation and the sustainable industrial production of high-quality *P. praeruptorum*.

## Glossary

ABC: ATP-binding cassette
ABF: ABA-responsive element binding factor
ARR: arabidopsis response regulator
AS: angelicin synthase
ATG: autophagy-related gene
AUX/IAA: auxin/indole-3-acetic acid protein
BAHD: BAHD-type acyltransferase family
bHLH: basic helix-loop-helix
bZIP: basic leucine zipper transcription factor
BIN2: brassinosteroid insensitive 2
BMT: bergaptol O-methyltransferase
C2’H: *p*-coumaroyl-CoA 2’-hydroxylase
C3’H: *p*-coumarate 3’-hydroxylase
C4H: cinnamate 4-hydroxylase
CA2H: caffeic acid 2-hydroxylase
CAL1: cauliflower 1
CAL2: cauliflower 2
CCoAOMT: caffeoyl-CoA O-methyltransferase
CCR: cinnamoyl-CoA reductase
CDF: cycling dof factor
CHS: chalcone synthase
4CL: 4-coumarate CoA ligase
CO: constans
COMT: caffeic acid O-methyltransferase
COMT-S: caffeic acid O-methyltransferase-similar
COP1: constitutive photomorphogenic 1
COSY: O-hydroxycinnamoyl-CoA-dependent coumarin synthase
CRY: cryptochrome
CSE: caffeoyl shikimate esterase
CYCD3: cyclin D3
CytC: cytochrome c
DC: decursinol cyclase
DELLA: DELLA protein
DMS: demethylsuberosin
ERF1: ethylene response factor 1
F6’H: feruloyl-CoA 6’-hydroxylase
FD: flowering locus D
FKF1: flavin-binding, kelch repeat, F-box 1
FLC: flowering locus C
FT: flowering locus T
GA: gibberellins
GI: gigantean
GID1: gibberellin insensitive dwarf 1
GID2: gibberellin insensitive dwarf 2
GIS: geographic information systems
H₂O₂: hydrogen peroxide
HCT: hydroxycinnamoyl-CoA shikimate transferase
HPLC: high-performance liquid chromatography
HS-GC-IMS: headspace gas chromatography-ion mobility spectrometry
ISSR: inter-simple sequence repeat
JAZ: jasmonate zim-domain protein
LFY: leafy
LHY: late elongated hypocotyl
MaxEnt: maximum entropy
MDR: multidrug resistance
MeJA: methyl jasmonate
MFT: mother of FT and TFL1
MP: monopteros
miR156: microRNA156
MS: mass spectrometry
MSI: mass spectrometry imaging
MYC2: myelocytomatosis 2
NAC: no apical meristem, arabidopsis transcription activation factor 1/2, and cup-shaped cotyledon 2
NIRS: near-infrared spectroscopy
NMR: nuclear magnetic resonance
OC: osthenol cyclase
OMT: O-methyltransferase
OPLS-DA: orthogonal partial least squares-discriminant analysis
P450: cytochrome P450
PAL: phenylalanine ammonia-lyase
PAP1: production of anthocyanin pigment 1
PCA: principal component analysis
PEBP: phosphatidylethanolamine-binding protein
PHY: Phytochrome
PIF3: phytochrome-interacting factor 3
POD: peroxidase activity
PRX: peroxidase gene
PS: psoralen synthase
PT: Prenyltransferases
R2R3-MYB: R2R3-myeloblastosis transcription factor family
SNRK2: sucrose non-fermenting 1-related protein kinase 2
SOC1: suppressor of overexpression of constans 1
SOD: superoxide dismutase
SPL: squamosa promoter-binding protein-like
SRAP: sequence-related amplified polymorphism
SSR: simple sequence repeat
TFL1: terminal flower 1
TOC1: timing of cab expression 1
U6PT: umbelliferone 6-prenyltransferase
U8PT: umbelliferone 8-prenyltransferase
UGT: UDP-glycosyltransferase
UV: Ultraviolet
XMT: xanthotoxin O-methyltransferase
ZTL: Zeitlupe
