# Supplementary material for: Quality formation in Peucedanum praeruptorum dunn: metabolite biosynthesis, geoherbal variation, and early bolting regulation
Source: Front Plant Sci. 2026 Apr 28;17:1812786. doi: 10.3389/fpls.2026.1812786 (PMC13161050; doi:10.3389/fpls.2026.1812786)
Supplement: Supplementary file 1 [file Table1.docx]

Supplementary Table 1 Main Coumarins identified from *P. praeruptorum*

| Classification | No. | Chemical Components | Formula | CAS No. | Analytical Methods | Pharmacological activities | Content range (mg/g) | References |
| --- | --- | --- | --- | --- | --- | --- | --- | --- |
| Simple coumarins | 1 | Apiosylskimmin | C_20_H_24_O_12_ | 103529-94-8 | NMR, MS | Antiplasmodial | NA | Ishii et al., 2008 |
|  | 2 | Eleutheroside B1 | C_17_H_20_O_10_ | 16845-16-2 | NMR, MS | Anti-inflammatory, antiviral | NA | Zhang et al., 2009 |
|  | 3 | Hymexelsin | C_21_H_26_O_13_ | 117842-09-8 | NMR, MS | NA | NA | Ishii et al., 2008 |
|  | 4 | Isofraxidin | C_11_H_10_O_5_ | 486-21-5 | NMR, MS | Anti-inflammatory, antioxidant, neuroprotective effect, cardioprotective effect, anticancer, anti-osteoporotic | 0.000-0.150 | Ishii et al., 2008 |
|  | 5 | Isoscopoletin | C_10_H_8_O_4_ | 776-86-3 | NMR, MS | NA | NA | Zhang et al., 2011 |
|  | 6 | Osthenol | C_14_H_14_O_3_ | 484-14-0 | HPLC | Anti-inflammatory, antioxidant, anticancer | 0.000-0.191 | Chen et al., 2021 |
|  | 7 | Praeroside VI | C_20_H_26_O_10_ | 1093132-23-0 | NMR, MS | NA | NA | Ishii et al., 2008 |
|  | 8 | Scopoletin | C_10_H_8_O_4_ | 92-61-5 | MS, IR, NMR | Anti-inflammatory, antioxidant, neuroprotective, anticancer, antimicrobial, antidiabetic, hepatoprotective | NA | Kong et al., 1994b |
|  | 9 | Scopolin | C_16_H_18_O_9_ | 531-44-2 | MS | Anti-inflammatory, anti-rheumatic, anti-obesity, antioxidant, neuroprotective, hepatoprotective | NA | Chen et al., 2019 |
|  | 10 | Skimmin | C_15_H_16_O_8_ | 93-39-0 | NMR, MS | Anti-inflammatory, renoprotective, antifibrotic, antioxidant, gastroprotective | NA | Okuyama et al., 1989 |
|  | 11 | Umbelliferone | C_9_H_6_O_3_ | 93-35-6 | NMR, MS | Anti-inflammatory, antioxidant, antidiabetic, antitumor, hepatoprotective, renoprotective, neuroprotective, antiepileptic, cardioprotective | 0.006-0.136 | Zhang et al., 2011 |
|  | 12 | 8-carboxy-7-hydroxy coumarin | C_11_H_8_O_5_ | 5112-55-0 | NMR, MS | NA | NA | Ishii et al., 2008 |
|  | 13 | (-)-peucedanol | C_15_H_18_O_5_ | 28095-18-3 | NMR, MS | Anti-inflammatory | NA | Kong et al., 1993a |
| Linear furanocoumarins | 1 | Arnocoumarin | C_14_H_10_O_3_ | 11037-15-3 | NMR, MS, HPLC | NA | NA | Chang and Li, 1999b |
|  | 2 | Bergapten | C_12_H_8_O_4_ | 484-20-8 | MS, HPLC | Anti-inflammatory, antimicrobial, antifungal, antiviral, anticancer, photosensitizing, anti-osteoporotic, anti-vitiligo | 0.000-0.471 | Chen et al., 2019 |
|  | 3 | Imperatorin | C_16_H_14_O_4_ | 482-44-0 | MS, HPLC | Anti-inflammatory, neuroprotective, anticancer, antidepressant | 0.000-1.188 | Chen et al., 2019 |
|  | 4 | Isopimpinellin | C_13_H_10_O_5_ | 482-27-9 | MS, HPLC | Anticonvulsant, photosensitizing | NA | Chen et al., 2019 |
|  | 5 | Isorutarin | C_20_H_24_O_10_ | 53846-51-8 | NMR, MS, HPLC | NA | NA | Okuyama et al., 1989 |
|  | 6 | Marmesin | C_14_H_14_O_4_ | 13849-08-6 | NMR, MS, HPLC | Anticancer | 0.000-0.074 | Okuyama et al., 1989 |
|  | 7 | Marmesinin | C_20_H_24_O_9_ | 27497-13-8 | NMR, MS, HPLC | Cardioprotective, antioxidant | NA | Chen et al., 2021 |
|  | 8 | Marmesin-11-O-β-D-glucopyranosyl-1→6-β-D-glucopyranoside | C_26_H_34_O_14_ | 1384983-00-9 | NMR, MS, HPLC | NA | NA | Wang et al., 2018 |
|  | 9 | Nodakenetin | C_14_H_14_O_4_ | 495-32-9 | NMR, MS | Anti-inflammatory, anti-osteoporotic, antiseizure, analgesic | NA | Asahara et al., 1984 |
|  | 10 | Nodakenetin tiglate | C_19_H_20_O_5_ | 106974-21-4 | NMR, MS, HPLC | NA | NA | Liu et al., 2014 |
|  | 11 | Nodakenin | C_20_H_24_O_9_ | 495-31-8 | MS | Anti-inflammatory, expectorant, antitussive, antiasthmatic, neuroprotective, antioxidant, antidepressant, anxiolytic | NA | Chen et al., 2019 |
|  | 12 | Oxypeucedanin | C_16_H_14_O_5_ | 737-52-0 | NMR, MS | Anti-inflammatory, anti-influenza, antitumor, antiallergic, antiseizure, analgesic | 0.002-0.340 | Zhang et al., 2011 |
|  | 13 | Oxypeucedanin hydrate | C_16_H_16_O_6_ | 133164-11-1 | NMR, MS | Anti-inflammatory, antiseizure, antibacterial, antiproliferative, anti-melanogenic | NA | Zhang et al., 2011 |
|  | 14 | Peucedanoside B | NA | NA | NMR, MS, HPLC | NA | NA | Chang et al., 2007 |
|  | 15 | Praeroside I | C_28_H_30_O_13_ | 121064-73-1 | NMR, MS, HPLC | NA | NA | Liu, 2020 |
|  | 16 | Praeroside VII | NA | NA | NMR, HPLC | NA | NA | Chang et al., 2008 |
|  | 17 | Psoralen | C_11_H_6_O_3_ | 66-97-7 | MS | Photosensitizing, antitumor, antibacterial, antifungal, antiviral, anti-osteoporotic | 0.000-0.058 | Jian et al., 2020 |
|  | 18 | Qianhucoumarin G | C_14_H_14_O_5_ | 68692-61-5 | IR, NMR, MS | NA | NA | Ling-Yi et al., 1996 |
|  | 19 | Rutaretin | C_14_H_14_O_5_ | 13895-92-6 | NMR, MS, HPLC | NA | NA | Chen et al., 2021 |
|  | 20 | Xanthotoxin | C_12_H_8_O_4_ | 298-81-7 | MS | Anti-inflammatory, anticonvulsant, photosensitizing, neuroprotective, procognitive, antidepressant, antitumor, antiparkinsonian, anti-vitiligo | 0.000-0.265 | Chen et al., 2019 |
|  | 21 | Rutarin | C_20_H_24_O_10_ | 20320-81-4 | NMR, MS, HPLC | NA | NA | Okuyama et al., 1989 |
| Angular furanocoumarins | 1 | Angelicin | C_11_H_6_O_3_ | 523-50-2 | MS | Anti-inflammatory, antitumor, antimicrobial | NA | Jian et al., 2020 |
|  | 2 | Apterin | C_20_H_24_O_10_ | 53947-89-0 | NMR, MS, HPLC | Anti-inflammatory | NA | Chang et al., 2007 |
|  | 3 | Oroselol | C_14_H_12_O_4_ | 1891-25-4 | NMR, MS | Antitumor | NA | Wang et al., 2018 |
|  | 4 | Peucedanoside A | C_20_H_22_O_10_ | 946122-87-8 | NMR, MS, HPLC | NA | NA | Chang et al., 2007 |
|  | 5 | Sphondin | C_12_H_8_O_4_ | 483-66-9 | NMR, MS | Anti-inflammatory | NA | Zhang et al., 2011 |
| Linear pyranocoumarins | 1 | Aegelinol | C_14_H_14_O_4_ | 21860-31-1 | NMR, HPLC | Antibacterial, aldose reductase inhibitory | NA | Chang and Li, 1999a |
|  | 2 | Decursitin D | C_19_H_20_O_6_ | 245446-61-1 | NMR, MS | NA | NA | Zhang et al., 2005 |
|  | 3 | Decursinol angelate | C_19_H_20_O_5_ | 130848-06-5 | NMR, MS, HPLC | Anti-inflammatory, anti-metastatic, antitumor, antidiabetic | NA | Liu, 2020 |
| Angular pyranocoumarins | 1 | D-laserpitin | C_19_H_20_O_6_ | 134002-17-8 | NMR, MS | NA | NA | Lee et al., 2015 |
|  | 2 | Hyuganin D | C_20_H_22_O_7_ | 77331-76-1 | MS, HPLC | NA | NA | Chen et al., 2019 |
|  | 3 | Khellactone | C_14_H_14_O_5_ | 518-76-3 | MS, HPLC | NA | NA | Chen et al., 2019 |
|  | 4 | Praeruptorin A | C_21_H_22_O_7_ | 73069-25-7 | NMR, MS, HPLC | Anti-inflammatory, antiasthmatic, anticancer, expectorant, analgesic, antithrombotic | 1.517-27.397 | Xiong et al., 2012 |
|  | 5 | Praeruptorin B | C_24_H_26_O_7_ | 81740-07-0 | MS, HPLC | Analgesic, antithrombotic, anticancer | 0.202-15.028 | Chen et al., 2019 |
|  | 6 | Praeruptorin C | C_24_H_28_O_7_ | 83382-71-2 | NMR | Anti-inflammatory, anti-osteoporotic, expectorant, neuroprotective, analgesic, antithrombotic, anticancer, antitussive | NA | Chen et al., 1979 |
|  | 7 | Praeruptorin D | C_24_H_26_O_7_ | 73069-26-8 | NMR | Anti-inflammatory, analgesic, antithrombotic, anticancer | NA | Chen et al., 1979 |
|  | 8 | Praeruptorin E | C_24_H_28_O_7_ | 78478-28-1 | MS, HPLC | Anti-inflammatory, analgesic, anticancer | 0.106-7.687 | Chen et al., 2019 |
|  | 9 | Peucedanocoumarin I | C_21_H_24_O_7_ | 130464-55-0 | NMR, MS, HPLC | NA | NA | Liu, 2020 |
|  | 10 | Peucedanocoumarin II | C_21_H_22_O_7_ | 130464-56-1 | NMR, MS, HPLC | Induction of plant disease resistance | 0.000-1.703 | Takata et al., 1990 |
|  | 11 | Peucedanocoumarin III | C_21_H_22_O_7_ | 130464-57-2 | NMR, MS, HPLC | Anti-aggregation, neuroprotective | NA | Takata et al., 1990 |
|  | 12 | Praeroside II | C_20_H_24_O_10_ | 86940-46-7 | NMR, MS, HPLC | NA | NA | Takata et al., 1988 |
|  | 13 | Praeroside III | C_20_H_24_O_10_ | 117306-98-6 | NMR, MS, HPLC | NA | NA | Takata et al., 1988 |
|  | 14 | Praeroside IV | C_20_H_24_O_10_ | 117233-34-8 | NMR, MS, HPLC | NA | NA | Takata et al., 1988 |
|  | 15 | Praeroside V | C_20_H_24_O_9_ | 117233-35-9 | NMR, MS, HPLC | NA | NA | Takata et al., 1988 |
|  | 16 | Pteryxin | C_21_H_22_O_7_ | 13161-75-6 | NMR, MS, HPLC | Antitumor | 0.000-5.531 | Liu, 2020 |
|  | 17 | Qianhucoumarin A | C_19_H_20_O_6_ | 150135-35-6 | MS, HPLC | NA | 0.000-0.112 | Chen et al., 2019 |
|  | 18 | Qianhucoumarin B | C_16_H_16_O_6_ | 152615-14-0 | MS, HPLC | NA | NA | Chen et al., 2019 |
|  | 19 | Qianhucoumarin C | C_16_H_16_O_6_ | 152615-15-1 | NMR, MS, HPLC | NA | NA | Liu, 2020 |
|  | 20 | Qianhucoumarin D | C_18_H_18_O_7_ | 20516-19-2 | MS, HPLC | NA | 0.017-2.546 | Chen et al., 2019 |
|  | 21 | Qianhucoumarin E | C_19_H_18_O_6_ | 156041-02-0 | MS, HPLC | NA | NA | Chen et al., 2019 |
|  | 22 | Selinind | C_19_H_20_O_5_ | 19427-82-8 | NMR, MS | NA | NA | Lee et al., 2015 |
|  | 23 | Suksdorfin | C_21_H_24_O_7_ | 53023-17-9 | NMR, MS | Anti-HIV | NA | Lee et al., 2015 |
|  | 24 | (-)-trans-khellactone | C_14_H_14_O_5_ | 23458-04-0 | NMR, MS, HPLC | NA | NA | Kong et al., 1993b |
|  | 25 | (+)-cis-khellactone | C_14_H_14_O_5_ | 24144-61-4 | NMR, MS, HPLC | NA | NA | Kong et al., 1993b |

Note: NA, data not available or not reported in the referenced literature.

Supplementary Table 2 Volatile Oils identified from *P. praeruptorum*

| Classification | No. | Chemical Components | Formula | CAS No. | Analytical Methods | References |
| --- | --- | --- | --- | --- | --- | --- |
| Volatile oils | 1 | α-farnesene | C_15_H_24_ | 502-61-4 | MS, GC | Yu et al., 2007 |
|  | 2 | α-pinene | C_10_H_16_ | 2437-95-8 | MS, GC | Yu et al., 2007 |
|  | 3 | Aromadendrene | C_15_H_24_ | 109119-91-7 | MS, GC | Yu et al., 2007 |
|  | 4 | Hinokitol | C_10_H_12_O_2_ | 499-44-5 | MS, GC | Yu et al., 2007 |
|  | 5 | Longifolene | C_15_H_24_ | 475-20-7 | MS, GC | Yu et al., 2007 |
|  | 6 | Terpinolene | C_10_H_16_ | 586-62-9 | NMR, MS, GC | Yu et al., 2007 |

Supplementary Table 3 Flavonoids identified from *P. praeruptorum*

| Classification | No. | Chemical Components | Formula | CAS No. | Analytical Methods | References |
| --- | --- | --- | --- | --- | --- | --- |
| Flavonoids | 1 | 4H-1-benzopyran-4-one, 5-hydroxy-6-methoxy-2-phenyl-7-O-α-D-glucuronyl acid | C_22_H_20_O_11_ | 36948-76-2 | NMR, MS, HPLC | Zhang et al., 2012 |
|  | 2 | 4H-1-benzopyran-4-one, 5-hydroxy-6-methoxy-2-phenyl-7-O-α-D-glucuronyl methyl ester | C_23_H_22_O_11_ | 82475-02-3 | NMR, MS | Zhang et al., 2012 |

Supplementary Table 4 Organic Acids identified from *P. praeruptorum*

| Classification | No. | Chemical Components | Formula | CAS No. | Analytical Methods | References |
| --- | --- | --- | --- | --- | --- | --- |
| Organic acids | 1 | Vanillic acid | C_8_H_8_O_4_ | 121-34-6 | NMR, MS | Kong et al., 1994a |
|  | 2 | Gallic acid | C_7_H_6_O_5_ | 149-91-7 | NMR, MS | Kong et al., 1994a |
|  | 3 | Butyric acid | C_4_H_8_O_2_ | 107-92-6 | NMR, MS | Zhang et al., 2009 |
|  | 4 | Palmitic acid | C_16_H_32_O_2_ | 57-10-3 | NMR, MS | Zhang et al., 2006 |
|  | 5 | Tetracosanoic acid | C_24_H_48_O_2_ | 302912-17-0 | NMR, MS | Zhang et al., 2006 |
|  | 6 | 9,10-dihydrophenanthrinic acid | C_17_H_10_O_7_ | 1239614-84-6 | UV, IR, MS | Zhang et al., 2010 |
